# Supplementary figures and images for: Quantitation of Cellular Dynamics in Growing Arabidopsis Roots with Light Sheet Microscopy
Source: PLoS One. 2011 Jun 22;6(6):e21303. doi: 10.1371/journal.pone.0021303 (PMC3120859; doi:10.1371/journal.pone.0021303)

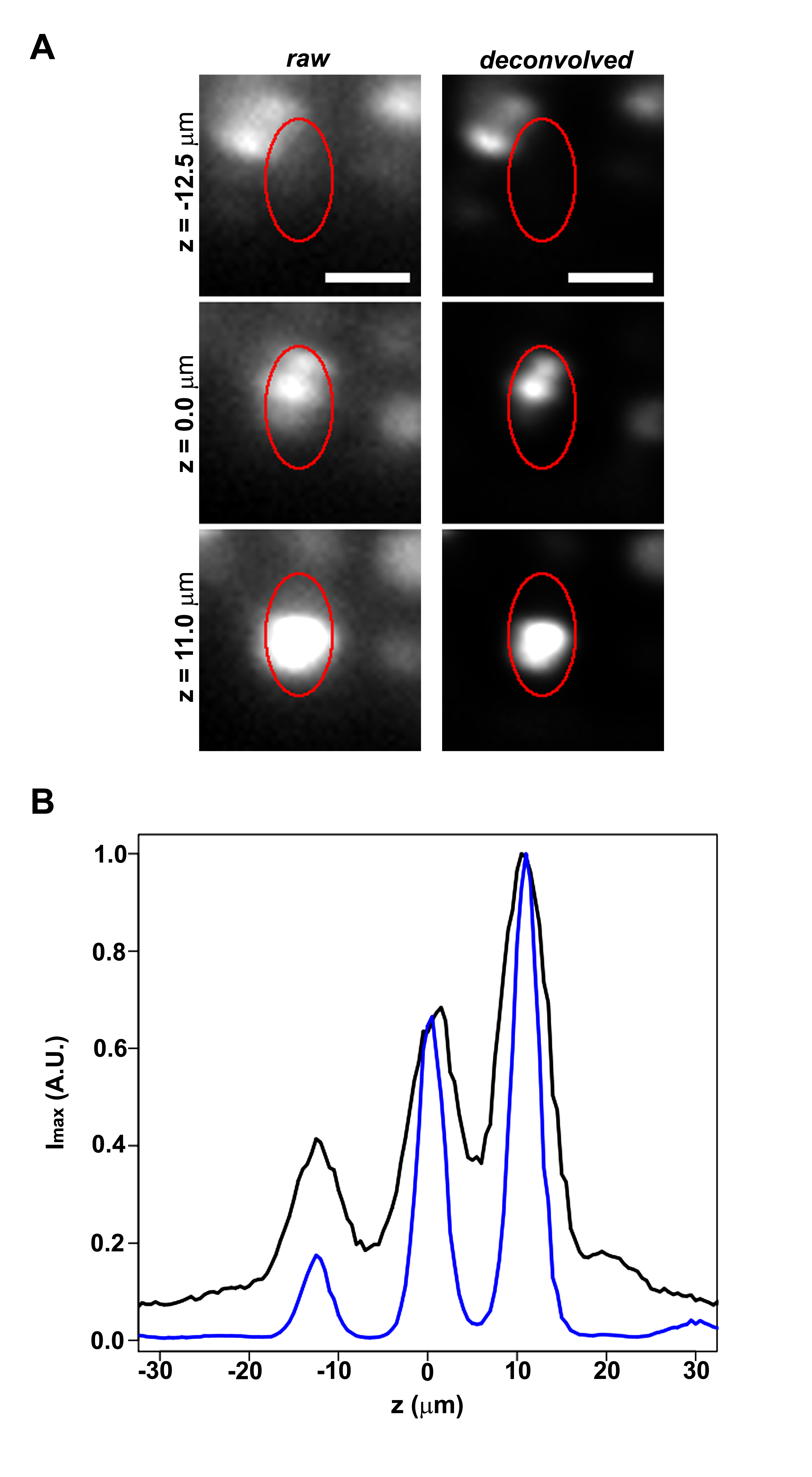

Supplement: Figure S1 — Effects of deconvolution in the axial ( z ) dimension. (A) Raw (left column) and deconvolved (right column) images of three close nuclei, at different z-positions; bar, 7.5 µm. (B) Maximum intensity collected in the region marked by the red ellipses in (A), both for the raw (black) and the deconvolved (blue) images. The increased resolution in z after deconvolution is shown by the improved separation between peaks. (TIF) [file pone.0021303.s001.tif]

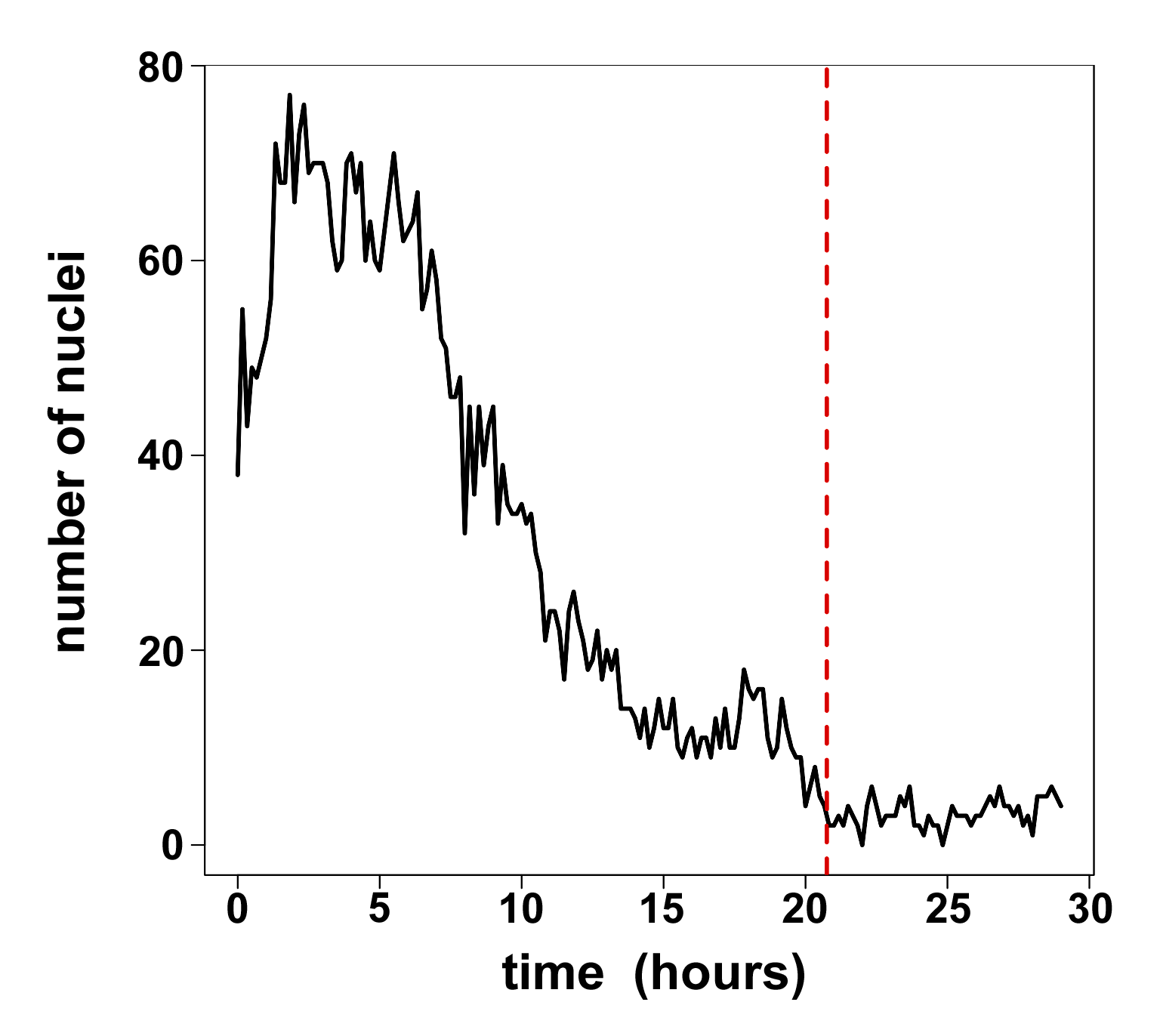

Supplement: Figure S2 — Contraction of the root radius. Number of nuclei with radial coordinate ρ greater than 55 µm, as a function of time. The red dotted line indicates the time point (21 hours) chosen to distinguish “early” and “late” temporal windows. (TIF) [file pone.0021303.s002.tif]

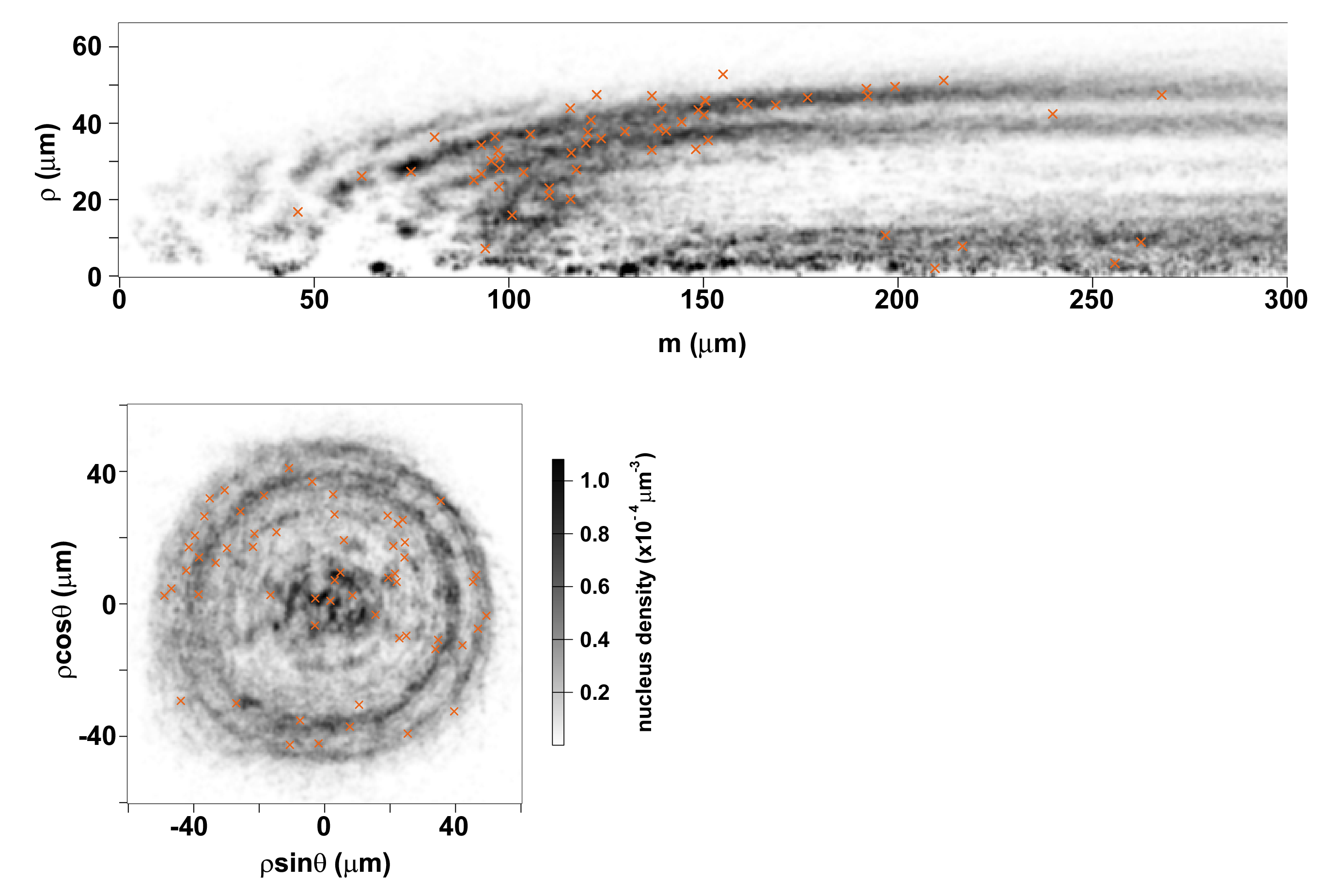

Supplement: Figure S3 — False positives. Positions of the false positive cell divisions found in the same dataset containing the true positives shown in Fig. 5 of the main text, superimposed on the smoothed density estimate of all the nuclei segmented at all time points throughout the same 29 hour interval. (TIF) [file pone.0021303.s003.tif]

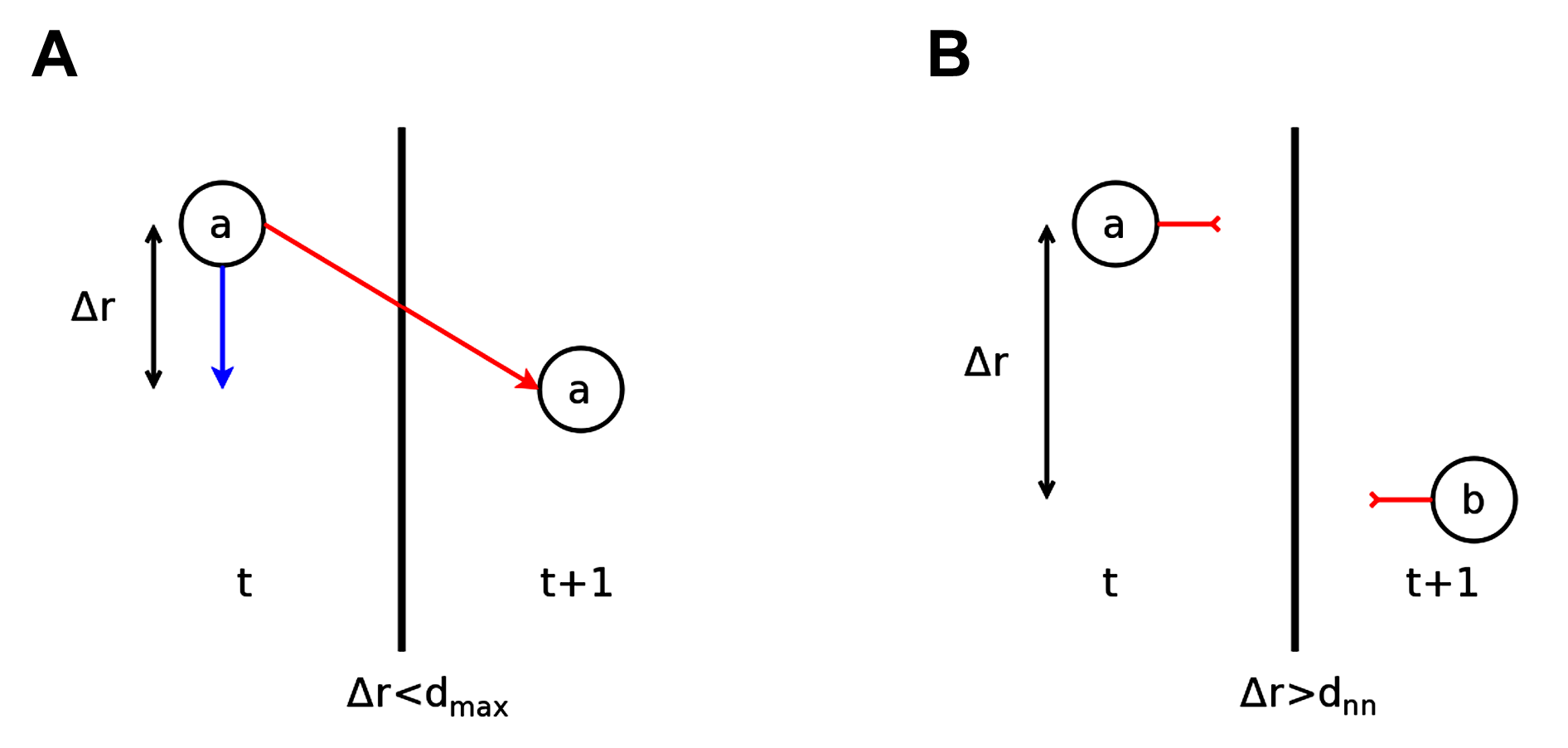

Supplement: Figure S4 — Tracking nuclei: example. Diagram showing the correct assignments in two typical scenarios (A and B) for two consecutive time-points (t and t+1). (A) The same nucleus a is observed at both times t and t+1, with a spatial displacement (blue arrow): the two nuclei should be assigned to the same trajectory (link between them, red arrow). (B) Nucleus a is only observed at time t and nucleus b is only observed at time t+1: the two nuclei should not be assigned to the same trajectory (no link between them, red truncated links). Δr, distance between nuclei observed in t and t+1; d max = 5 µm, maximum nucleus displacement observed between t and t+1 (time-step = 10 min); d nn = 10 µm, average distance between nearest neighbor nuclei. (TIF) [file pone.0021303.s004.tif]
