# Supplementary material for: Quantitation of Cellular Dynamics in Growing Arabidopsis Roots with Light Sheet Microscopy
Source: PLoS One. 2011 Jun 22;6(6):e21303. doi: 10.1371/journal.pone.0021303 (PMC3120859; doi:10.1371/journal.pone.0021303)
Supplement: Table S1 — Performance of segmentation and tracking routines. Segmentation: error rates in nuclei recognition in the regions I–V defined in figure 2C; N, number of manually identified nuclei; FP, false positive rate; FN, false negative rate. Tracking: error rates in nucleus-trajectory assignment in the same regions I–V; N, number of manually identified trajectories starting in the given region; error range, lower and upper bounds for errors in assigning a nucleus to the correct trajectory. (PDF) [file pone.0021303.s005.pdf]

Table S1

	Segmentation			***Tracking***	
	**N**	**FP (%)**	**FN (%)**	**N**	**Error range (%)**
**I**	255	1.6	9.4	18	1.3 – 2.4
**II**	238	3.8	9.2	16	3.9 – 5.6
**III**	220	11.4	5.9	15	2.3 – 3.8
**IV**	239	1.7	5.0	19	1.4 – 2.4
**V**	351	15.1	5.4	32	2.8 – 4.6
**TOT**	**1303**	**7.3**	**6.9**	**100**	**2.4 – 3.9**
